# Prion protein self-peptides modulate prion interactions and conversion

**DOI:** 10.1186/1471-2091-10-29

**Published:** 2009-11-30

**Authors:** Alan Rigter, Jan Priem, Drophatie Timmers-Parohi, Jan PM Langeveld, Fred G van Zijderveld, Alex Bossers

**Affiliations:** 1 Department of Bacteriology and TSEs, Central Veterinary Institute (CVI) of Wageningen UR, Lelystad, 8200 AB, the Netherlands; 2 Pepscan Presto BV, Lelystad, 8243 RC, The Netherlands

## Abstract

**Background:**

Molecular mechanisms underlying prion agent replication, converting host-encoded cellular prion protein (PrP^C^) into the scrapie associated isoform (PrP^Sc^), are poorly understood. Selective self-interaction between PrP molecules forms a basis underlying the observed differences of the PrP^C ^into PrP^Sc ^conversion process (agent replication). The importance of previously peptide-scanning mapped ovine PrP self-interaction domains on this conversion was investigated by studying the ability of six of these ovine PrP based peptides to modulate two processes; PrP self-interaction and conversion.

**Results:**

Three peptides (octarepeat, binding domain 2 -and C-terminal) were capable of inhibiting self-interaction of PrP in a solid-phase PrP peptide array. Three peptides (N-terminal, binding domain 2, and amyloidogenic motif) modulated prion conversion when added before or after initiation of the prion protein misfolding cyclic amplification (PMCA) reaction using brain homogenates. The C-terminal peptides (core region and C-terminal) only affected conversion (increased PrP^res ^formation) when added before mixing PrP^C ^and PrP^Sc^, whereas the octarepeat peptide only affected conversion when added after this mixing.

**Conclusion:**

This study identified the putative PrP core binding domain that facilitates the PrP^C^-PrP^Sc ^interaction (not conversion), corroborating evidence that the region of PrP containing this domain is important in the species-barrier and/or scrapie susceptibility. The octarepeats can be involved in PrP^C^-PrP^Sc ^stabilization, whereas the N-terminal glycosaminoglycan binding motif and the amyloidogenic motif indirectly affected conversion. Binding domain 2 and the C-terminal domain are directly implicated in PrP^C ^self-interaction during the conversion process and may prove to be prime targets in new therapeutic strategy development, potentially retaining PrP^C ^function. These results emphasize the importance of probable PrP^C^-PrP^C ^and required PrP^C^-PrP^Sc ^interactions during PrP conversion. All interactions are probably part of the complex process in which polymorphisms and species barriers affect TSE transmission and susceptibility.

## Background

Transmissible spongiform encephalopathies (TSEs) are fatal neurodegenerative disorders characterized by accumulation of the pathological isoform of prion protein mainly in tissues of the central nervous system. Formation of this pathological isoform is a posttranslational process and involves refolding (conversion) of the host-encoded prion protein (PrP^C^) into a pathological isoform partially protease resistant PrP^Sc ^(derived from scrapie) or PrP^res ^(PK-resistant PrP) [1]. The molecular mechanisms involved in PrP^C ^to PrP^Sc ^conversion are poorly understood, but polymorphisms in both PrP isoforms have been shown to be of importance in both interspecies and intraspecies transmissibilities [2]. The formation of PrP^Sc ^aggregates probably requires self-interactions of PrP^C ^molecules as well as with PrP^Sc ^[3,4]. Thus binding and conformational changes are essential events in this conversion process. Cell-free conversion of PrP^C ^provides a valuable *in vitro *model in which relative amounts of produced PrP^res ^reflect important biological aspects of TSEs at the molecular level [5,6]. A recent and very sensitive *in vitro *conversion system is the protein misfolding cyclic amplification (PMCA) assay [7-10], which has been shown to amplify minute amounts of PrP^Sc ^from a variety of sources including sheep scrapie [10]. The effects of single polymorphisms and species-barriers in PrP^C ^or PrP^Sc ^on PrP conversion can largely explain differences in susceptibility -and transmissibility in sheep scrapie [5,11-13]. Even though these polymorphisms are involved in modulation of disease development they do not seem to affect the initial binding of PrP^C ^to PrP^Sc ^[14] and do not seem to directly modulate PrP^C^-PrP^Sc ^binding. Furthermore, in a recent peptide-array mapping study of ovine PrP^C ^we concluded that these polymorphisms are not part of the identified PrP binding domains likely to be involved in PrP self-interaction [15]. However, this does not exclude these polymorphisms from posing indirect effects on binding behaviour of PrP^C ^to PrP^Sc ^and other possible chaperoning molecules. In that peptide-array binding study we unequivocally demonstrated that ovine PrP binds with PrP derived (self) amino acid sequences (sequence specific) separate from the polymorphic scrapie susceptibility determinants [15]. It remains to be elucidated whether the determined amino acid sequences play a role prior or during conversion in the self-interaction of PrP^C ^molecules and/or in the interactions of PrP^C ^with PrP^Sc^. Simultaneously, whether these amino acid sequences play a role in the processes underlying PrP conversion needs to be elucidated. In the current study we selected several ovine PrP sequence derived synthetic peptides to study not only their capacity to affect PrP binding to a solid-phase (PrP) peptide-array but also their potential modulating effect on PrP^C ^to PrP^Sc ^conversion.

## Results

**Figure 1:**
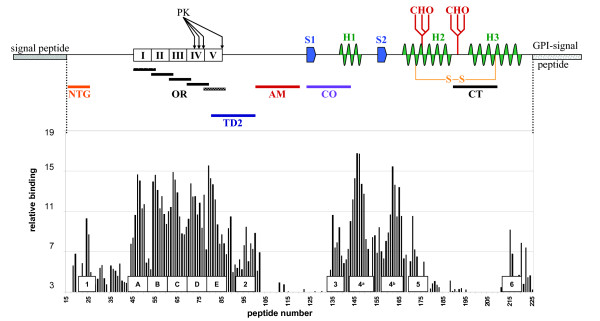
**PrP^C ^secondary structures and relative peptide positions versus peptide-array binding pattern and binding domain positions**. Schematic representation of PrP^C ^showing signal sequences, β-sheets (S1, S2), α-helices (H1, H2, H3), disulfide bridge (S-S), glycosylation sites (CHO) and the relative positions of the peptides used in this study. The bar graph represents the previously determined peptide-array binding pattern [15] and the relative positions of the determined interaction domains: **1 **[27-RPKPGGG-33], **A-E **octarepeat motif [PxGG], **2 **[102-WNK-104], **3 **[140-PLIHFGNDY-148], **4**^a ^[152-YYR-154], **4**^b ^[165-YYR-167], **5 **[177-NFV-179] and **6 **[225-SQAY-228]. All numbering used is for sheep PrP.

Previously we determined that recombinant ovine PrP yielded a reproducible sequence specific binding pattern with amino acid sequences using a solid-phase array of overlapping 15-mer peptides encompassing the complete ovine -or bovine amino acid sequence (peptide-array). Roughly this pattern breaks down into two high binding areas containing two-and three consensus domains respectively, combined with some lower binding domains (Figure 1). Based on the interaction domains extrapolated from this binding pattern as well as properties reported in literature, the following six ovine PrP regions were selected for peptide blocking studies. The sequences of these peptides represented structural properties of PrP as explained hereafter (summarized in table 1 and mapped in figure 1): **Peptide NTG**, spanning the amino acids (AAs) at the N-terminal part of the mature PrP^C^, including the glycosaminoglycan (GAG) binding motif KKRPK [16] and binding domain 1 [27-RPKPGGG-33] (ovine numbering used throughout, [15] and this study); **Peptide OR**, spanning the octarepeat AA motif [QPHGGGWG, AA 54-94] of the N-terminal region (PrP self-interaction was mapped to AA motif P(H)GG [15]), which is probably involved in a range of interactions [17-31] of which metal-binding is the best characterized; **Peptide TD2**, which overlaps the limiting region containing strain and species dependant variable sites for proteinase K trimming of PrP^Sc ^[32,33] and spanning binding domain 2 ([102-WNK-104], ovine numbering used throughout) of the first high binding area [15]; **Peptide AM**, which includes the amyloidogenic motif (AGAAAAGA) of PrP that did not exhibit any binding in the peptide-array [15]; **Peptide CO**, encompassing amino acids of the core region of PrP spanning from the first β-sheet onto the first α-helix. The peptide includes binding domain 3 [140-PLIHFGNDYE-149] and is immediately adjacent to binding domain 4^a ^of the second high binding area [15]; These domains are also important in PrP^Sc ^conformation-specific immuno-precipitation [34-40]; and **peptide CT**, spanning the C-terminal AA's covering part of the third helix, partially covering low binding domain 6 [192-TTTTKGENFT-202] and almost identical to a peptide capable of inhibiting cell-free conversion [41].

**Table 1:** Peptide information and peptide-array blocking results

peptide	amino acid sequence	position^1^	block^2^	**equiv**.^3^
**NTG**	KKRPKPGGGWNT	25-36	+/-	403

**OR**	GQPHGGGWGQ	61-95	++	396

**TD2**	GGGGWGQGGSHSQWNKPSK	89-107	++	198

**AM**	KTNMKHVAGAAAAGA	109-123	+/-	502

**CO**	LGSAMSRLPLIHFGNDYEDR	133-151	no	499*

**CT**	GENFTETDIKIMERVVEQMC	198-217	++	198

^1 ^position of the amino acid sequence in mature ovine PrP^C^

^2 ^effect of pre-incubation with peptide on binding of PrP to the peptide-array

(-- no effect, +/- moderate blocking, ++ blocking)

^3 ^minimal amount of molar excess of peptide needed to affect PrP binding

(* highest amount of excess peptide tested)

### PrP peptide inhibition of PrP self-binding to peptide-array

First these six peptides were tested for their capability to inhibit PrP binding to the PrP based peptide-array containing 242 peptides (15-mer) overlapping each other by increments of 1 AA, covering the complete ovine PrP amino acid sequence (results summarized in Table 1).

**Figure 2:**
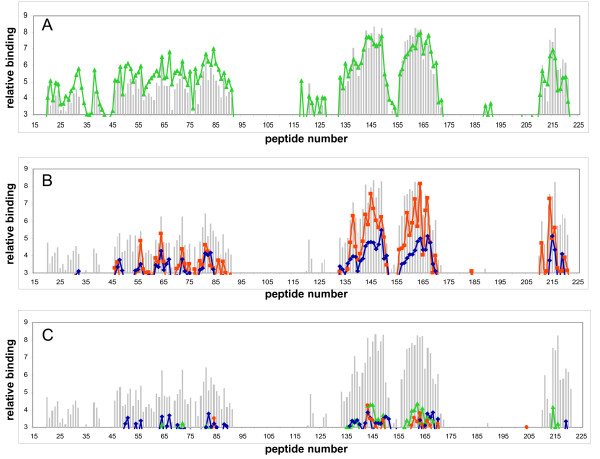
**Effects of peptides on PrP to peptide-array binding pattern**. No effect (A) on the peptide-array binding pattern and intensities was observed when PrP was pre-incubated with peptide CO (green line) compared to non-peptide pre-incubated PrP binding (grey bars). Only moderate inhibition of the binding pattern (B) was observed when PrP was pre-incubated with either peptide NTG (blue line) or peptide AM (orange line), whereas almost complete inhibition was observed when PrP was pre-incubated with either peptide OR (blue line), TD2 (green line) or CT (orange line). Minimal peptide concentration needed for blocking was determined (Table 1).

Pre-incubation of PrP with peptide CO did not result in blocking of the binding pattern of PrP on the peptide-array (Figure 2a), whereas peptides NTG and AM only moderately blocked the binding pattern of PrP (Figure 2b). Peptide NTG seems to diminish binding throughout the binding pattern (Figure 2b, blue line), whilst peptide AM mainly affects binding with the peptides derived from the N-terminal part of mature PrP (Figure 2b, green line). Maximum blocking throughout the PrP binding pattern occurred with peptides OR (Figure 2c, blue line), TD2 (Figure 2c, green line) and CT (Figure 2c, orange line), which all block equally throughout the PrP binding pattern. However, in contrast to blocking studies performed with antibodies [15], blocking was not absolute over the whole region of the PrP peptide-array binding pattern. Inhibition by the aforementioned peptides was dose-dependant, with maximum blocking only occurring when peptides were added at high molecular ratios to PrP. Pre-incubation of PrP with (at least) 400 times molar excess of peptide OR or (at least) 200 times molar excess of peptides TD2 and CT was necessary to obtain maximum blocking of the PrP binding pattern on the peptide-array.

The capability of the N-terminal peptide NTG to moderately block binding of PrP to the peptide-array and also moderately modulated PrP^res ^formation in the supplemented -and pre-incubated PMCA assay (described below) necessitates re-evaluation of the previously determined consensus domain [33-GWNTG-37] [15]. Instead of this common consensus domain two binding domains seem present in the N-terminus. Binding motifs determined by motif-grafted antibodies [42] suggests that the domain of interest is [27-RPKPGGG-33], which encompasses most of the proposed glycosaminoglycan binding motif [25-KKRPK-29] [16].

These results confirm the importance of the previously mapped domains [15] located within the first high binding area [PHGG] (octarepeat) and [102-WNK-104], as well as the importance of the C-terminal low binding domain [192-TTTTKGENFT-202] in PrP self-interaction. To a lesser extent the involvement of the N-terminal glycosaminoglycan binding motif contained within domain [27-RPKPGGG-33] and amyloidogenic motif [116-AGAAAAGA-123] in interaction is confirmed. Interestingly peptide CO, encompassing the previously mapped domain [140-PLIHFGNDYE-149] did not influence binding of PrP to the peptide-array.

### PrP peptides modulation of PrP^res ^formation in the PMCA-assay

The peptides analyzed in the prion protein peptide-array were also studied for their modulating capacity in the sheep PrP protein misfolding cyclic amplification (PMCA) assay [9,10,43] using sheep brain homogenates from confirmed scrapie-positive and scrapie-negative sheep in one round of sonication cycles. To test the influence of the peptides on conversion, peptides were either added after combining the scrapie positive -and negative brain homogenates (peptide supplemented PMCA) or alternatively peptide was added first to the scrapie-negative brain homogenate before addition of the scrapie-positive material (peptide pre-incubated PMCA). This allowed us to assess if the effect of the peptides on conversion was dependant on the first rapid interaction between PrP^C ^and PrP^Sc ^[14] or not. Peptide was added in several molar ratios, relative to the calculated total amount of PrP^C ^present in the reaction. PrP^Sc ^specific proteinase-K (PK) resistant fragments were quantified by Western blotting.

### Peptide supplemented PMCA

**Figure 3:**
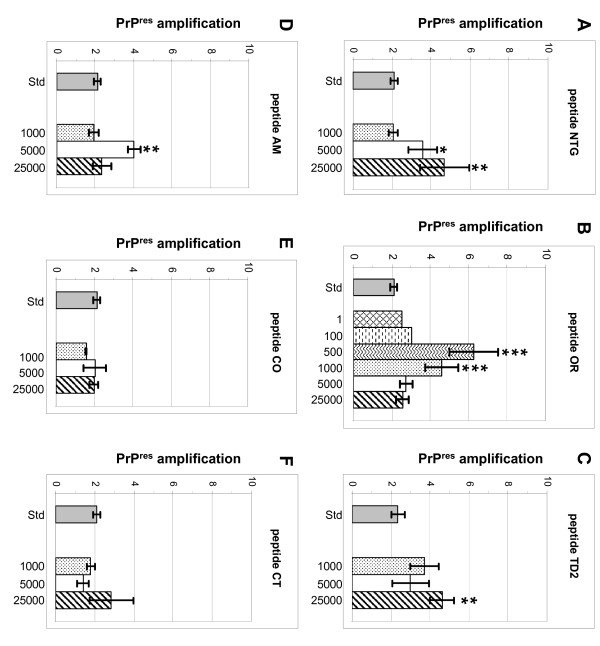
**Effects of peptide supplementation on PrP^res ^formation in ovine PMCA**. Bar graphs depicting PrP^res ^formation of a standard PMCA reaction compared to a PMCA reaction supplemented with different molar excess amounts of peptide NTG (A), peptide OR (B), peptide TD2 (C), peptide AM (D), peptide CO (E) and peptide CT (F). For each peptide its specific corresponding standard is depicted (grey bar). In order to determine the optimal molar excess of octarepeat peptide OR in the supplemented PMCA, a single test with peptide OR added at molar excess 1, 10, 100 and 250 was performed. The amounts of PrP^res ^in these reactions were comparable to the standard. As an example results at molar excess 1 and 100 are depicted in graph B. The number of independent measurements (n), the median and s.e.m. for each peptide and molar excess are summarized in Table 2. An unpaired Student's t-test was performed to determine whether PrP^res ^formation with peptide was significantly different to the corresponding standard PrP^res ^formation. P-values are listed in Table 2 and significant differences are marked; p-values between 0.05 and 0.001 with *, p-values between 0.001 and 0.0001 with ** and p-values = 0.0001 with ***.

Addition of peptide after mixing scrapie positive -and negative brain homogenates resulted in a dose dependant increase of PK resistant PrP (PrP^res^) after sonication for four [NTG, OR, TD2 and AM] of the six peptides tested (results summerized in Table 2). In general the amount of newly formed PrP^res ^roughly ranged between 2-fold (standard reaction) up to 8-fold as compared to the input amount of PrP^Sc^. Addition of a large molar excess of peptide NTG (Figure 3a) resulted in a significant increase in PrP^res ^formation in a dose dependant manner at molar excesses of 5.000 (p = 0.0161) and 25.000 (p = 0.0007). Addition of peptide OR (Figure 3b) resulted in the largest increase of PrP^res^. However, in contrast to the other peptides the effect was inversely dose dependant and significant at molar excesses of 500 (p < 0.0001) and 1000 p = 0.0001). Molar excesses below 500 of peptide OR did not result in significant increase of PrP^res ^as with higher molar excess of this peptide. Addition of peptide TD2 (Figure 3c) resulted in a slight to moderate increase in PrP^res ^formation. PrP^res ^increase was significant only at a molar excess of 25.000 (p = 0.0027). Addition of peptide AM (Figure 3d) resulted in a moderate and significant increase of PrP^res^, but only when peptide was added at a molar excess of 5.000 (p = 0.0012). Addition of either peptide CO (Figure 3e) or CT (Figure 3f) did not significantly influence PrP^res ^formation at the tested molar excesses. However it does seem that these peptides slightly inhibit PrP^res ^formation at molar excesses of 1000 and 5000 respectively.

### Peptide pre-incubated PMCA

**Figure 4:**
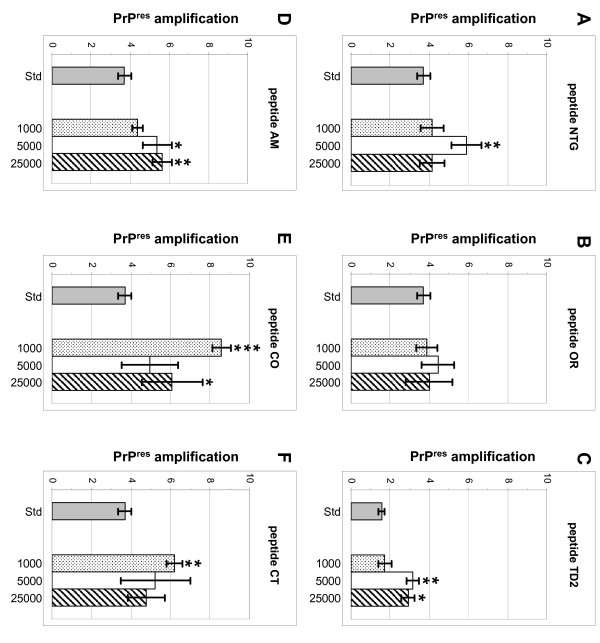
**Effects of peptide pre-incubation on PrP^res ^formation in ovine PMCA**. Bar graphs depicting PrP^res ^formation of a standard PMCA reaction compared to a PMCA reaction in which the scrapie negative homogenate was pre-incubated with peptide with different molar excess amounts of peptide NTG (A), peptide OR (B), peptide TD2 (C), peptide AM (D), peptide CO (E) and peptide CT (F) before adding scrapie positive homogenate. For each peptide its specific corresponding standard is depicted (grey bar). The number of independent measurements (n), the median and s.e.m. for each peptide and molar excess are summarized in Table 2. An unpaired Student's t-test was performed to determine whether the PrP^res ^formation with peptide was significantly different to the corresponding standard PrP^res ^formation. P-values are listed in Table 2 and significant differences are marked; p-values between 0.05 and 0.001 with *, p-values between 0.001 and 0.0001 with ** and p-values ≤ 0.0001 with ***.

Pre-incubating scrapie-negative brain homogenate with peptide before initiating conversion with scrapie-positive brain homogenate, surprisingly resulted in increased formation of proteinase-K resistant PrP (PrP^res^) after sonication for five [NTG, TD2, AM, CO and CT] of the six peptides tested (results summerized in Table 2) generally in a dose dependant manner. However, compared to the peptide supplemented PMCA assay results differed for several of the peptides. Pre-incubation with peptide NTG (Figure 4a) resulted in a slight but significant increase of PrP^res ^at the optimal molar excess of 5.000 (p = 0.0076). In contrast to peptide supplemented PMCA reactions, pre-incubation with peptide OR (Figure 4b) did not significantly affect PrP^res ^formation at any of the molar excesses tested. Pre-incubation with peptide TD2 (Figure 4c) induced a moderate but significant increase of PrP^res ^at molar excesses 5.000 (p = 0.0048) and 25.000 (p = 0.0113), with an apparent optimum at 5.000. Whereas peptide AM induced amplification of PrP^res ^optimally at molar excess 5.000 in the supplemented PMCA assay, pre-incubation with this peptide (Figure 4d) resulted in a slight but significant dose dependant increase in PrP^res ^at molar excesses 5.000 (p = 0.0362) and 25.000 (p = 0.0044). Pre-incubation peptide CO (Figure 4e) resulted in a significant moderate or slight increase in PrP^res ^at molar excesses 1.000 (p < 0.0001) and 25.000 (p = 0.0496). However, increase of PrP^res ^at molar excess of 1.000 was larger and unmistakably more significant than at the higher molar excess, suggesting an inverse dose dependant increase of peptide induced PrP^res ^formation. Finally, pre-incubation of SNH with peptide CT (Figure 4f), also resulted in a significant inverse dose dependant increase of PrP^res ^at the molar excess of 1.000 (p = 0.0006).

**Table 2:** Peptide modulation of supplemented -and pre-incubated PMCA assay

		complemented PMCA-assay				pre-incubated PMCA-assay			
peptide	**m.e**.^1^	median	**s.e.m**.	n^2^	**sign**.^3^	median	**s.e.m**.	n^2^	**sign**.^3^
**NTG**	1000	2.07	0.24	3		4.15	0.60	5	
	5000	3.56	0.73	3	*	5.91	0.76	5	**
	25000	4.68	1.26	3	**	4.13	0.63	5	

**OR**	500	6.26	1.27	4	***	^§^*n.t*.			
	1000	4.61	0.87	5	***	3.85	0.54	4	
	5000	2.74	0.68	4		4.44	0.85	4	
	25000	2.57	0.33	2		3.99	1.19	4	

**TD2**	1000	3.71	0.73	8	n.q.	1.73	0.35	4	
	5000	3.00	0.93	8		3.15	0.33	4	**
	25000	4.63	0.62	8	**	2.91	0.34	4	*

**AM**	1000	1.92	0.27	3		4.36	0.28	7	
	5000	4.03	0.34	3	**	5.38	0.76	7	*
	25000	2.36	0.46	3		5.63	0.50	7	**

**CO**	1000	1.56	0.02	2		8.59	0.46	5	***
	5000	2.02	0.58	2		4.95	1.43	5	
	25000	1.97	0.22	2		6.09	1.53	5	*

**CT**	1000	1.80	0.21	2		6.21	0.39	5	**
	5000	1.41	0.29	2		5.23	1.76	5	
	25000	2.85	1.12	2		4.77	0.92	5	

^1 ^amount of molar excess of peptide compared to PrP^C ^tested in the PMCA assay

^2 ^numbers of independent measurements performed

^3 ^comparison of conversion ratios of the peptide supplemented -or pre-incubated PMCA and their corresponding standards using the unpaired Student's t-test (p-values < 0.05 are considered statistically different). Significantly different values are marked; p-values between 0.05 and 0.001 with *, p-values between 0.001 and 0.0001 with ** and p-values ≤ 0.0001 with ***. The p-value of a comparison that was not quite significantly different is marked with n.q.

^§^n.t. = not tested

### PMCA assay negative controls

Even though each PMCA assay setup revealed at least one PrP specific peptide incapable of modulating PrP^res ^formation, additional negative controls were performed. To rule out the possibility that factors other than PrP sequence specificity could be responsible for the observed results, the isoelectric point, net charge and average hydrophilicity were determined for each peptide (data not shown). Comparison revealed no clear correlation between these non-sequential features and the observed effects in the PMCA assay. Therefore the following additional negative controls were performed; In order to determine whether addition of just a random peptide is sufficient for modulating PrP^res ^formation an unrelated peptide (canine parvo virus specific sequence peptide DGAVQPDGGQPAVRNER) was used in both testing setups described above. Addition of this peptide did not affect PrP^res ^formation in either of the two PMCA assay setups (data not shown) at various concentrations, indicating that the observed increases in PrP^res ^were a result of the specific PrP derived peptide amino acid sequences added to the reactions. Furthermore, PMCA assays were also performed for each peptide without scrapie positive homogenate, to determine whether *de novo *PrP^res ^could be formed when the PrP derived peptide was combined with PrP^C^. Peptide was added at the optimum (in the supplemented PMCA) molar excess for peptide NTG (1.000), OR (500) and AM (5.000). While peptides TD2, CO and CT were added at the highest molar excess (25.000) used in the in the PMCA assays described above. No significant conversion induced by either of these peptides was detected after PK digestion (data not shown), showing that only addition of scrapie-positive brain homogenate resulted in initiation of the conversion reaction.

## Discussion

**Figure 5:**
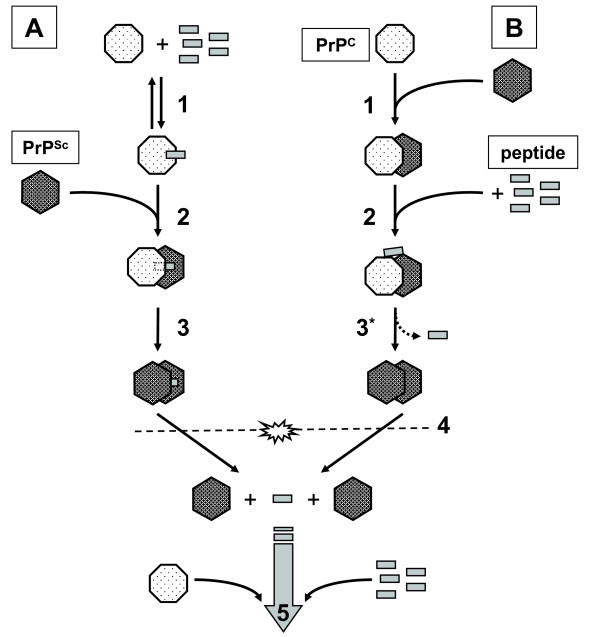
**Schematic representation of one incubation/sonication cycle of PMCA**. Peptide is allowed to form a bond with PrP^C ^(1), resulting in all PrP^C ^binding peptide(s) (pre-incubation with an excess of peptide). The conversion reaction is initiated by adding PrP^Sc ^to the 'sensitized' PrP^C ^(2). It is possible that the peptide remains associated with the formed PrP^res ^during conversion (3). Sonication (4) shears PrP^res^, but does not necessarily release peptide from 'sensitized' PrP^C ^and probably also not from the elongated PrP^Sc ^(#). The PrP^res ^fragments are in turn capable of recruiting and converting 'sensitized' PrP^C ^(5) during the next incubation cycle.

**Figure 6:**
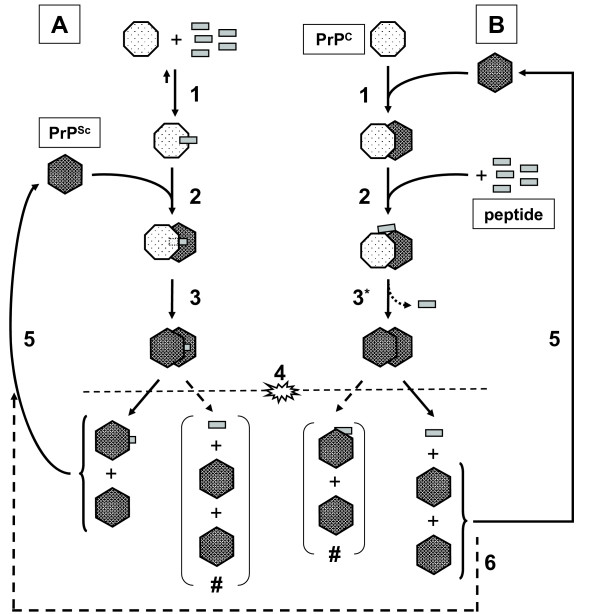
**Adapted schematic representation of one incubation/sonication cycle of PMCA. *Pre-incubated PMCA *(A)**: peptide is allowed to form a bond with PrP^C ^(1), resulting in all PrP^C ^binding peptide (pre-incubation with an excess of peptide). The conversion reaction is initiated by adding PrP^Sc ^to the 'sensitized' PrP^C ^(2). It is likely that the peptide is incorporated in formed PrP^res ^during conversion (3). Sonication (4) shears PrP^res^, but does not release peptide from 'sensitized' PrP^C ^and probably not from the elongated PrP^Sc ^(#). The PrP^res ^fragments (some with peptide incorporated) are in turn capable of recruiting and converting 'sensitized' PrP^C ^(5) during the next incubation cycle. ***Supplemented PMCA *(B)**: the conversion reaction is first initiated by combining PrP^C ^and PrP^Sc ^(1). Peptide is immediately added (2), which can bind to either PrP^C ^or PrP^Sc ^separately, but is probably only effective when binding both. PrP^C ^is converted, possibly releasing the peptide in the process (3). Sonication (4) shears PrP^res^, probably releasing the peptide if it is still bound after step 3. The PrP^res ^fragments (unlikely with peptide incorporated, #) are in turn capable of recruiting and converting PrP^C ^(5) during the next incubation cycle. Even though PrP^C^, PrP^res ^and peptide are all present after sonication, it is likely that fist the PrP^C^-PrP^Sc ^complex is formed (1) before interaction with peptide (2) occurs. However, if the peptide is capable of 'sensitizing' PrP^C^, the reaction proceeds as described for the pre-incubated PMCA reaction (6). After addition of all ingredients in the first cycle the pre-incubated -and supplemented PMCA reactions will have both unbound and bound peptide available for the following sonication-incubation cycles.

In a previous study [15] we showed that ovine PrP binds to itself and mapped several domains using ovine (and bovine) prion protein derived peptide-arrays, yielding a PrP-specific binding pattern for soluble (monomeric) PrP that could be blocked by several PrP-specific monoclonal antibodies. The current study shows that the different PrP derived synthetic peptides exert different effects on binding in a peptide-array assay and on conversion of PrP^C ^to PrP^res ^in the PMCA assay. In order to better interpret the PMCA data, one needs to consider the effects of sonication in the PMCA. It may be expected that after the first incubation cycle, sonication simply results in shearing the elongated PrP^Sc ^which releases the peptide (Figure 5). This results in multiple seeds for the following incubation cycle so that after the first sonication cycle, conditions for both the pre-incubated and the supplemented PMCA can be considered identical. This is however not in agreement with the obtained results (discussed below). Therefore we have devised a more intricate schematic (Figure 6) to better account for the observed differences between the pre-incubated (Figure 6A) and supplemented PMCA (Figure 6B) for some of the peptides.

**Figure 7:**
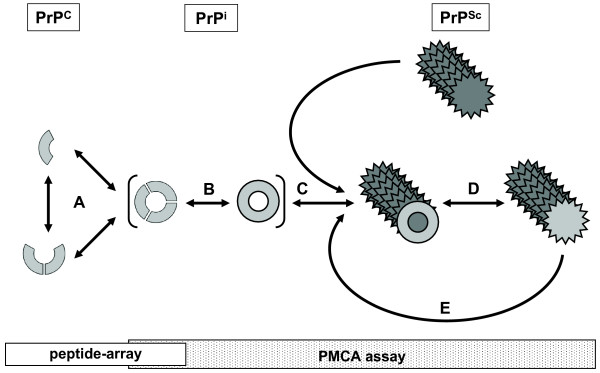
**Schematic representation of likely steps in prion protein conversion**. Preceding conversion a multimer of PrP^C ^molecules is formed [A]. Possibly a dimer is formed and both monomeric -and dimeric PrP is partially refolded before forming the structural subunit of a fibril; trimeric PrP. PrP^C ^may however first form a trimer before partially unfolding and/or refolding. Partial unfolding/refolding is at the basis of forming an intermediate isoform of PrP (PrP^i^) [B]. PrP^i ^can subsequently interact with the conversion seed (PrP^Sc^) [C], which allows conversion of PrP into PrP^Sc^. Several PrP^i ^may have to be 'stacked' onto the conversion seed before conversion of the first PrP^i ^bound can occur. During conversion PrP^i ^is further refolded into PrP^Sc^, its tertiary structure adapted to that of the conversion seed (D). The newly elongated PrP^Sc ^in turn can act as a seed for further conversion of PrP (E). The bars beneath the schematic denote for which conversion processes the peptide-array results (white bar) and PMCA assay results (shaded bar) are indicative.

The conversion process is a succession of distinct steps, which most likely starts with the multimerisation of PrP^C^. Wille *et al*. used electron crystallography to characterize the structure of two infectious variants of the prion protein [44]. By comparing projection maps of these two variants a model featuring β-helices was devised. This model was further refined by studying 119 all-β-folds observed in globular proteins [3]. It was proposed that PrP^Sc ^should adopt a β-sandwich, parallel β-helical architecture, or a parallel left-handed β-helical fold. This left-handed β-helical folded PrP can readily form trimers, providing a natural template for a trimeric model of PrP^Sc ^and another (similar) β-helical model was proposed, which largely explained species and strain-specificity [4]. In both models oligomerisation/trimerisation of PrP^C ^precedes initiation of conversion as depicted in Figure 7A, implying an important role for PrP^C ^self-interaction in the conversion processes. Our data fits this multimerisation of PrP^C^. Additionally, study of the amyloid-forming pathway revealed a pre-amyloid state containing partially unfolded monomers and dimers (PrP^i^) [45-50]. Whether PrP^i ^is just the partially unfolded state (monomers and dimers) of PrP^C ^or whether it is a pre-formed trimer before further structural rearrangement towards PrP^Sc ^occurs (Figure 7B) remains to be elucidated. Conversion is initiated by recruitment of PrP to PrP^Sc ^(Figure 7C), after which PrP is (further) rearranged to adopt the tertiary structure of the PrP^Sc ^seed (Figure 7D). The elongated PrP^Sc ^is in turn capable to recruit and convert further PrP (Figure 7E). Taking these studies into account and their implications for conversion allows for a more detailed interpretation of the data presented in this study., Herein the peptide-array data is indicative for the effects on soluble PrP (PrP^C^-PrP^C ^interaction), whereas the PMCA assay may be indicative for effects on PrP interactions (self- and PrP^C^-PrP^Sc ^interaction) as well as interactions with chaperoning or inhibiting molecules. All six PrP-derived peptides tested affected conversion in either the supplemented and/or pre-incubated PMCA assay, whereas binding to the peptide-array was only completely abolished by three peptides [OR, TD2 and CT]. Taken together, this study shows that the previously determined self-interaction domains of PrP^C ^are of importance at several different phases in the conversion reaction.

Ovine peptide-array analysis previously revealed two high binding areas within PrP [15] of which the first high binding area encompasses the octarepeats, more specifically the consensus domain P(H)GG. This study showed that the octarepeat peptide (OR) was capable of blocking the binding pattern of PrP to the peptide-array, probably as a result of peptide-induced changes in the tertiary structure of the N-terminal tail and thus affecting PrP^C ^self-interaction. Only in the supplemented PMCA assay, a dose dependant and significant increase in PrP^res ^is observed. The octarepeats can modulate [51-55] but are not a necessity for the molecular processes underlying conversion [21,56]. Interaction between PrP^C ^and PrP^Sc ^seems almost instantaneous [14], which would leave the peptide free to interact with the PrP^C ^- PrP^Sc ^complex as a whole or with co-factors present in the homogenates. Because the octarepeat stabilizes the interaction of PrP^C ^with the LRP-LR receptor [57], it seems that peptide OR indirectly affects the conversion process, either by affecting PrP^i ^stability/formation (Figure 7B) or by stabilizing PrP^i ^interaction with PrP^Sc ^(Figure 7C). Furthermore, a di-peptide containing the octarepeat self-aggregates into nanometric fibrils [58] and these may also be formed in the supplemented PMCA assay. Combined with our data, we propose that the flexible N-terminal tail containing the octarepeat region stabilizes PrP^C^-PrP^Sc ^interaction during conversion and that free peptide OR forms nanometric fibrils mimicking and increasing PrP^C^-PrP^Sc ^stabilization, thus aiding subsequent conversion. Also part of the peptide-array first high binding area is binding domain 2 ([102-WNK-104], Figure 1) and the data presented here shows that pre-incubation of the peptide TD2 (containing [102-WNK-104]) with PrP abolished binding of PrP to the peptide-array. This indicates the importance of this domain in PrP^C ^self-interaction (Figure 7A and possibly 7B). Increased PrP^res^production was observed in both the supplemented -and the pre-incubated PMCA-assay. The mechanism by which the peptide stimulates PrP^res ^formation may simply be due to peptide enhanced interaction between separate PrP molecules. Alternatively, peptide TD2 could aid unfolding and/or refolding of PrP^C ^during conversion; binding domain 2 [102-WNK-104], together with the amyloidogenic motif, is part of the region of PrP^C ^that is partially unfolded and refolded during oligomerization of PrP^C ^into a β-sheet-rich soluble isoform of PrP [47].

The second high binding area in the peptide-array contains the domain [140-PLIHFGNDY-148] (domain 3, Figure 1). This study shows that the peptide CO containing the domain [140-PLIHFGNDY-148] does not affect binding of PrP to the peptide-array at all, thereby ruling out direct involvement in PrP^C ^self-interaction. Several studies have established that polymorphisms at sheep PrP amino acid position 136, 154 and 171 surrounding [140-PLIHFGNDY-148] are most relevant in differential TSE susceptibility [5,59-63] and that stability of this PrP region is a crucial determinant in whether PrP^C ^is converted [40,64] (affecting species-barrier and/or scrapie susceptibility). Therefore peptide CO induced PrP^res ^formation in the pre-incubated PMCA assay is either due to this core region peptide facilitating binding of PrP^C ^to PrP^Sc ^or the peptide affects the stability of this region of PrP^C ^(interacting with the 'self-domain' or another domain of PrP) thereby facilitating refolding of PrP.

The N-terminal peptide NTG (containing the glycosaminoglycan binding motif) only moderately affects binding of PrP^C ^throughout the peptide-array, suggesting that interaction of the peptide with PrP^C ^results either in slight changes in the tertiary structuring affecting solubility of PrP^C ^or in diminished availability of the previously determined domains [15] for interaction with the peptide-array. Intriguingly, peptide NTG induces PrP^res ^formation in both the supplemented (dose dependant) and pre-incubated (dose optimum) PMCA assay. The glycosaminoglycan heparan sulphate proteoglycan (HSPG) and pentosan polysulphate (PPS) stimulate PrP^res ^formation *in vitro *and suggests that free glycosaminoglycans acted as a contact-mediator allowing interaction of PrP^C ^and PrP^Sc ^[65]. The N-terminal peptide NTG likely indirectly affects *in vitro *conversion either by mimicking glycosaminoglycan binding to domain [27-RPKPGGG-33] or by recruiting glycosaminoglycans onto PrP^C^, facilitating conversion of PrP^C ^into new PrP^Sc ^after seeding. These studies and our PMCA assay data strongly implicate glycosaminoglycans as an important cofactor in the conversion process.

The ability of peptide AM, which encompasses the amyloidogenic motif [116-AGAAAAGA-123], to moderately block binding of PrP to the peptide-array was somewhat surprising, since we previously showed that the amyloidogenic motif was not involved in PrP self-interaction [15]. Peptide AM mainly inhibits binding of PrP to the peptides covering the N-terminal part of the mature PrP protein, suggesting that peptide AM interacts with one (or more) of the other previously determined binding domains. In contrast to earlier reports [41,66,67], we observed that peptide AM (containing the amyloidogenic motif) slightly but significantly increased PrP^res ^formation in both the pre-incubated -and supplemented PMCA. However, all these inhibiting peptides contained two or more additional amino acids of the putative aggregation sites (flanking the amyloidogenic motif) implicated in aggregation/oligomerisation [68], suggesting inhibition by these peptides is due to interference with aggregation/oligomerization. Additionally, differences between the used conversion systems (i.e. availability of cofactors) are likely to play a role as well. The peptide AM used in this study specifically focuses only on the amyloidogenic motif. Our data suggests that peptide AM interacts with the N-terminal tail of PrP^C ^(octarepeat motif or [102-WNK-104]), probably altering its tertiary structure and facilitating the proposed stabilizing effect of the N-terminal tail. Alternatively, peptides containing only the amyloidogenic motif are also capable of forming a β-sheet rich layer at the water-air interface when sonicated [69] and peptide AM may form a β-sheeted backbone that interacts with the PrP^C^-PrP^Sc ^complex, mimicking and/or complementing the proposed stabilizing effect of the N-terminal tail.

Peptide CT overlaps most of the third alpha helix of PrP^C ^as well as the second glycosylation site and the second cysteine involved in the di-sulphide bridge formed in PrP^C^. The capacity to completely block PrP binding to the peptide-array suggests that the domain [225-SQAY-228] is of importance in PrP^C ^self-interaction. This study shows a slight significant increase in PrP^res ^formation when peptide CT is pre-incubated with scrapie negative brain homogenate. This contradicts results using a similar peptide capable of inhibiting cell free conversion [41]. However, this inhibiting peptide is four amino acids larger than peptide CT, which may account for the difference in effects and/or it may just be due to the differences in experimental technique between the cell free conversion and the PMCA assay. This seems to be corroborated by the observation that in the supplemented PMCA (setup closest resembling conditions in cell free conversion [41]) peptide CT seems to slightly inhibit PrP^res ^formation albeit not significantly. Fibrillization of a human PrP peptide fragment is hindered by disulfide bridge formation between two peptides [70] or when an additional disulfide bridge is introduced [34], which indicates that peptide CT (when pre-incubated with PrP^C^) likely compromises the disulfide bridge, destabilizing PrP^C^, which consequently promotes trimerisation or formation of a conversion intermediate (Figure 7B) and thus facilitating conversion.

In the PMCA-assay all peptides revealed an inducing effect on PrP^res ^formation in the supplemented -and/or pre-incubated PMCA-assay. Above possible explanations for these effects have been discussed for each peptide. However it can not be ruled out that the peptides may have had an opposite effect; instead of interacting with PrP, peptide may have interacted with possible conversion inhibitory factors present in the homogenate, thus indirectly allowing conversion to take place more efficiently. Identifying these possible 'natural' inhibitory factors may prove an alternative line of investigation towards the underlying mechanisms involved in prion replication and may provide additional targets for future prion therapy.

## Conclusion

The binding domains found for ovine PrP^C ^using a prion protein peptide-array are primarily indicative of prion protein self-interaction. Apparently several specific self-interactions between individual PrP molecules occur, which include both PrP^C^-PrP^C ^as well as PrP^C^-PrP^Sc ^interactions. The data presented here imply an influence of binding domain [140-PLIHFGNDY-148] on the stability of the region of PrP previously determined to be involved in the species-barrier and/or susceptibility to scrapie. Furthermore our data indicates a stabilizing function for the octarepeats region (N-terminal tail) in PrP^C^-PrP^Sc ^interaction and thus improving subsequent conversion. Our data further suggests that the N-terminal glycosaminoglycan binding motif [27-RPKPGGG-33] affects the conversion process indirectly, and implicates glycosaminoglycans as an important cofactor in prion disease pathogenicity. Peptide AM containing the amyloidogenic motif indirectly affects conversion either by aiding and/or complementing the proposed stabilizing function of the N-terminal tail of PrP^C^. Finally, the data implicates direct involvement of the two binding domains [102-WNK-104] and [225-SQAY-228] in self-interaction between PrP^C ^molecules preceding binding to PrP^Sc ^and subsequent conversion. Therefore these two domains may prove prime targets for development of new therapeutic strategies. Our results emphasize the importance of the stability of the PrP^C^-PrP^C ^and PrP^C^-PrP^Sc ^interactions in PrP conversion, which is an essential determinant in the effects of disease associated mutations, as well as the species-barrier. Focussing on the (stabilizing) self-interaction domains of PrP and the subsequent conversion processes may lead to further therapeutic strategies with the possibility to leave the physiological function of the prion protein unaffected.

## Methods

### MBP-PrP construction, expression and purification

The mature part of sheep PrP (ARQ) open reading frame (ORF) was cloned into the pMAL Protein Fusion and Purification System (New England Biolabs) as described before [15], resulting in the maltose binding protein (MBP) fusion to the N-terminus of PrP (MBP-PrP). MBP-PrP was expressed and purified by affinity chromatography as described in the manual of the pMAL Protein Fusion and Purifications System (method I; New England Biolabs) To improve binding of MBP-PrP and to prevent formation of interchain disulfide upon lysis (as suggested in the protocol), β-mercaptoethanol was added. Quantity and quality of the eluted MBP-PrP was determined before use in the peptide-array by SDS-PAGE (12% NuPAGE, Invitrogen). After separation the gel was either stained with Sypro Orange (total protein stain, Molecular Probes) or analyzed by Western blotting and immunodetection of MBP-PrP with polyclonal antiserum R521-7 specific for PrP [71].

### Peptides

Peptides were synthesized with an acetylated N-terminus and an amidated C-terminus as described before [71]. The synthesized peptides were purified by high performance liquid chromatography using mass spectrometric analysis for identification. The resulting purified peptides were at least 90% pure. All peptides dissolved well in water and solutions were stored frozen. Sequential properties like iso-electric point were calculated with the Peptide Property Calculator made available online in the tools section of Innovagen http://www.innovagen.se.

### Peptide-array analysis

Synthesis of complete sets of overlapping 15-mer peptides were carried out on grafted plastic surfaces, covering the ovine PrP amino acid sequence of mature PrP (residues 25-234 [72]). Coupling of the peptides to the plastic surface consisting of a 455-well credit-card size plastic (minicard) and subsequent ELISA analyses including subsequent background correction, relative density value calculation and binding pattern interpretation were performed as described before [15,73]. This study also showed that linking of MBP to PrP did not have any disadvantageous effects and therefore its properties are indicative for PrP. Peptide blocking studies were performed by pre-incubating the MBP-PrP with molar excesses of prion peptides before incubating the PrP-peptide mixture as the antigen on the minicard. Binding to the peptide-array was considered relevant when at least at 3 consecutive peptides optical density values of at least 2.5 times the background were observed.

### Protein Misfolding Cyclic Amplification assay

The protein misfolding cyclic amplification (PMCA) assay first described by Saborio *et al*. [9] and has been shown applicable to amplify PrP^Sc ^from different sources [10,43]. In short; a 10% brain homogenate from a (confirmed) scrapie-positive sheep (SPH) was diluted with 50-100 times in 10% (confirmed) scrapie-negative sheep brain homogenate (SNH) after which the reaction was subjected to one round of sonication-incubation cycles (24 hours; 48 cycles). To test the influence of the peptides on conversion, the peptides were either added after combining the scrapie positive -and negative brain homogenates (supplemented PMCA) or peptide was pre-incubated with the scrapie-negative homogenate (pre-incubated PMCA) before addition of the scrapie-positive brain homogenate. The total amount of PrP^C ^in the PMCA reaction was calculated based on the quantification of the amount of PrP^C ^in brain tissue [74]. The amount of peptide needed for a reaction was calculated based on the total amount of PrP^C ^in the reaction, adjusted for the size difference between peptide and PrP^C^, so that the amount of peptide added represents various molar excesses of peptide molecules relative to the total number of PrP^C ^molecules present in the reaction. The amount of formed PrP^res ^was determined after proteinase K digestion (100 μg/ml) of the PMCA reaction and analysis by SDS-PAGE and subsequent Western blotting. Standard detection of PrP^res ^was performed with monoclonal antibody 9A2 [75], except for PMCA reactions containing peptide SAU14, which contains the binding epitope of 9A2. In this case we used a proven combination of monoclonal antibodies L42 [76] and Sha31 [77]. To determine whether the determined amounts of PrP^res ^were significantly different in comparison to its corresponding standard PMCA-assay reaction, an unpaired Student's t-test was performed and the p-value calculated.

## Abbreviations

TSE: transmissible spongiform encephalopathies; PrP: prion protein; PrP^C^: host encoded cellular prion protein; PrP^Sc^: disease associated prion protein; PrP^res^: proteinase K resistant prion protein; PMCA: protein misfolding cyclic amplification

## Authors' contributions

AR and AB conceived the study and together with JPML were responsible for study design and coordination. All PMCA assays were performed by JP and all peptide-arrays were performed at Pepscan presto B.V. by DPT. Supporting experiments and data-analysis were performed by AR. AR, JPML and AB were responsible for data interpretation. The experiments for the study were facilitated by grants under the supervision of AB, JPML and FGvZ. AR drafted the manuscript and AB and JPML critically read the manuscript before submission.
